# Plasmalemmal Vesicle Associated Protein-1 (PV-1) is a marker of blood-brain barrier disruption in rodent models

**DOI:** 10.1186/1471-2202-9-29

**Published:** 2008-02-26

**Authors:** Eveline H Shue, Eleanor B Carson-Walter, Yang Liu, Bethany N Winans, Zarina S Ali, Jun Chen, Kevin A Walter

**Affiliations:** 1 Department of Neurosurgery, University of Pittsburgh, Pittsburgh, PA 15213, USA; 2 Department of Neurosurgery, University of Rochester, Rochester, NY 14642, USA; 3 Department of Neurology, University of Pittsburgh, Pittsburgh, PA 15213, USA

## Abstract

**Background:**

*Plasmalemmal vesicle associated protein-1 *(*PV-1*) is selectively expressed in human brain microvascular endothelial cells derived from clinical specimens of primary and secondary malignant brain tumors, cerebral ischemia, and other central nervous system (CNS) diseases associated with blood-brain barrier breakdown. In this study, we characterize the murine CNS expression pattern of *PV-1 *to determine whether localized *PV-1 *induction is conserved across species and disease state.

**Results:**

We demonstrate that *PV-1 *is selectively upregulated in mouse blood vessels recruited by brain tumor xenografts at the RNA and protein levels, but is not detected in non-neoplastic brain. Additionally, *PV-1 *is induced in a mouse model of acute ischemia. Expression is confined to the cerebovasculature within the region of infarct and is temporally regulated.

**Conclusion:**

Our results confirm that *PV-1 *is preferentially induced in the endothelium of mouse brain tumors and acute ischemic brain tissue and corresponds to blood-brain barrier disruption in a fashion analogous to human patients. Characterization of *PV-1 *expression in mouse brain is the first step towards development of rodent models for testing anti-edema and anti-angiogenesis therapeutic strategies based on this molecule.

## Background

Vasogenic cerebral edema causes significant morbidity and mortality in patients with malignant brain tumors. Cytokines secreted by the growing tumor such as VEGF, PDGF, and SF/HGF increase the permeability of the blood-brain barrier (BBB) causing extracellular fluid accumulation. The increase in extracellular fluid, in turn, raises intracranial pressure leading eventually to brain ischemia, herniation, and death. The situation repeats itself in the setting of cerebral infarction where the initial intracellular swelling associated with cytotoxic edema is later compounded by cytokine release and vasogenic edema. Clinical attempts to block these cascades have focused on inhibiting signal transduction by the circulating cytokines, including monoclonal antibodies targeted to VEGF and selective receptor tyrosine kinase inhibitors. An equally attractive approach may be to identify selective targets on the endothelial cells themselves, which are associated with BBB alterations.

In our previous studies of blood-brain barrier breakdown and endothelial changes in malignant glioma, we have described several microvascular endothelial genes associated with BBB alterations. Using this approach, *PV-1 *was identified as a Glioma Endothelial Marker (GEM), a microvascular endothelial marker that is upregulated by glioma signaling [1-3]. *PV-1 *encodes a transmembrane protein that is associated with the caveolae of fenestrated microvascular endothelial cells. It is normally expressed in the lung, liver, kidney, and immature brain of rodents [4]. However, intracerebral expression of *PV-1 *is silenced during normal differentiation of the blood-brain barrier, where transendothelial transport is inhibited [5]. Preliminary work has demonstrated that *PV-1 *expression is not only upregulated in malignant brain tumors, but it is also induced in cases of acute ischemia/stroke [6]. Downregulation of *PV-1 *via *PV-1 *targeted siRNA, decreases endothelial tubule formation normally induced by Matrigel [6]. Therefore, we suggested that *PV-1 *upregulation may indicate a general state of blood-brain barrier disruption and endothelial activation, such as would be associated with malignancy, ischemia, or trauma.

We hypothesized that the expression of mouse *PV-1 *would parallel the expression of human *PV-1 *described previously. In the current study, we demonstrate upregulation of *PV-1 *protein expression in the mouse cerebrovasculature, using human glioblastoma (GBM) cell lines grown as intracranial xenografts. We also describe induction of *PV-1 *in a mouse model of acute ischemia, further expanding the role of *PV-1 *in states of blood-brain barrier disruption. These data confirm PV-1 expression as a marker of BBB dysfunction and should contribute to the generation of a rodent model for the development and preclinical testing of anti-angiogenic and anti-edema therapies targeted towards PV-1.

## Results and Discussion

### Expression of PV-1

**Figure 1:**
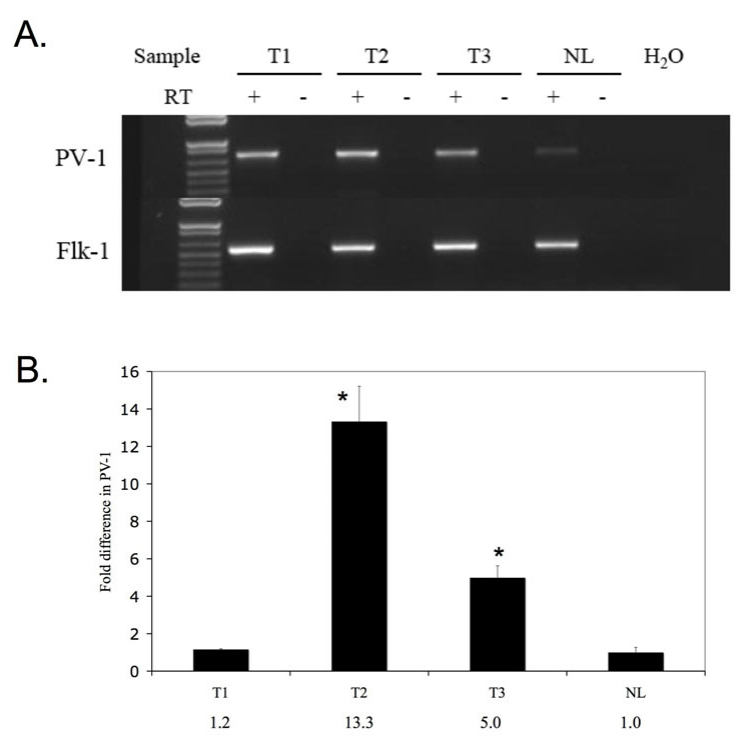
*PV-1 *mRNA expression in intracranial glioma xenografts. A. RT-PCR for *PV-1 *was performed on reverse-transcribed RNA harvested from U87MG tumors (T1) and U87:U87/VEGF tumors grown in mice (T2, T3) and compared to *PV-1 *expression levels in normal mouse brain (NL). Reactions were normalized using primers to the mouse endothelial gene, *flk-1*. B. Quantitative RT-PCR analysis of *PV-1 *expression. Reactions were normalized to *flk-1*. Numerical values for the fold increase are indicated. *Statistically significant difference between the levels of *PV-1 *expression in NL vs. T2 and NL vs. T3 (p = 0.05).

Previous studies in our laboratory have shown that *PV-1 *expression, normally silenced in non-neoplastic human brain, is upregulated in highly vascularized, human malignant brain tumors *in vivo *and is stimulated by VEGF *in vitro *[6]. We questioned whether mouse *PV-1 *expression would be induced in intracranial U87:U87/VEGF xenografts. The U87:U87/VEGF xenograft model is generated by injecting a 1:5 ratio of VEGF-overexpressing U87MG cells to untransfected U87MG cells [7] and personal communication]. Xenografts derived using this combination of cell lines demonstrate increased tumor vascularity, which facilitates detection of endothelial cells and small vessels, and better mimic the pathophysiology of human GBM tumors. RT-PCR was performed using murine specific *PV-1 *primers on mRNA isolated from standard U87MG mouse brain xenografts as well as U87:U87/VEGF xenografts. RT-PCR revealed that both xenograft tumor strains upregulated the expression of *PV-1 *in comparison to normal mouse brain. *Flk-1 *served as a positive control endothelial marker with comparable expression across all mouse brain samples (Figure 1A). Quantitative RT-PCR analysis of *PV-1 *expression in tumor versus normal tissues showed that while the U87MG tumors did consistently induce *PV-1 *RNA, the degree of RNA upregulation was several fold higher in the U87:U87/VEGF tumors (Figure 1B). Interestingly, we have had difficulty detecting *PV-1 *mRNA by *in situ *hybridization in standard, less vascular U87MG xenografts (data not shown), although we could detect protein upregulation by immunohistochemistry in the same sections, suggesting that VEGF may contribute to the increased expression of *PV-1 *mRNA as well as increasing tumor vascularity.

### Localization of PV-1 expression

**Figure 2:**
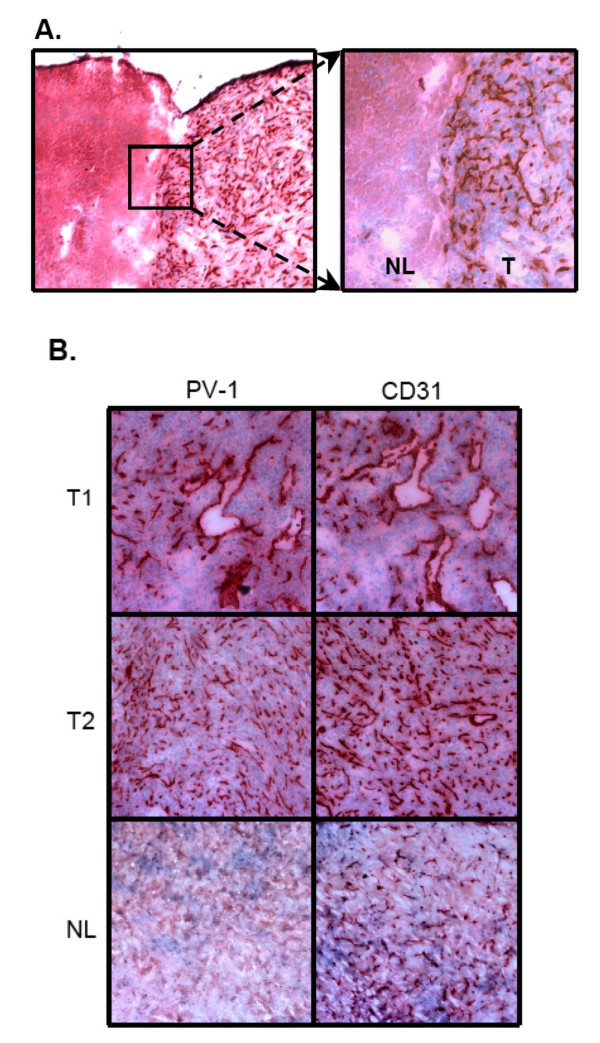
PV-1 protein expression in intracranial glioma xenografts. A. Immunohistochemistry for PV-1 was performed on frozen brain sections containing U87:U87/VEGF tumors (T1, T2) or normal brain (NL); 100×. PV-1 expression was detected solely on the microvessels within the tumors and showed a comparable staining pattern to that of the endothelial control marker, CD31. PV-1 expression was not detected in normal brain as compared to CD31. B. The interface between normal brain (NL) and brain tumor (T) demonstrated that PV-1 expression was tightly restricted to the tumor; overview 40×, detail 100×.

While *PV-1 *upregulation in mouse intracranial xenografts was detectable by RT-PCR, we wanted to confirm its induction and ensure that expression was localized to the tumor endothelium. Our studies have previously demonstrated that PV-1 colocalizes with endothelial control markers such as CD31 in human glioma vasculature [2]. Recent genomic data have demonstrated that mouse endothelial cell antigen 32 (meca-32) is identical to PV-1. The well-characterized monoclonal antibody, meca-32, was therefore used to detect PV-1, and the endothelial specific marker, CD31, was used as a positive control. Expression of PV-1 was strictly localized to the endothelium in the mouse brain tumors, and was consistently expressed throughout the tumor, from the core of the tumor to the tumor margin (Figures 2A, B). Non-neoplastic brain showed positive CD31 staining, but no PV-1 reactivity, indicating that glioma cells specifically induce the expression of PV-1 in mouse brain tumor endothelium (Figure 2B). Control tissues, which were not incubated with the primary antibodies, showed no endothelial PV-1 staining (data not shown).

### PV-1 expression in acute ischemia

**Figure 3:**
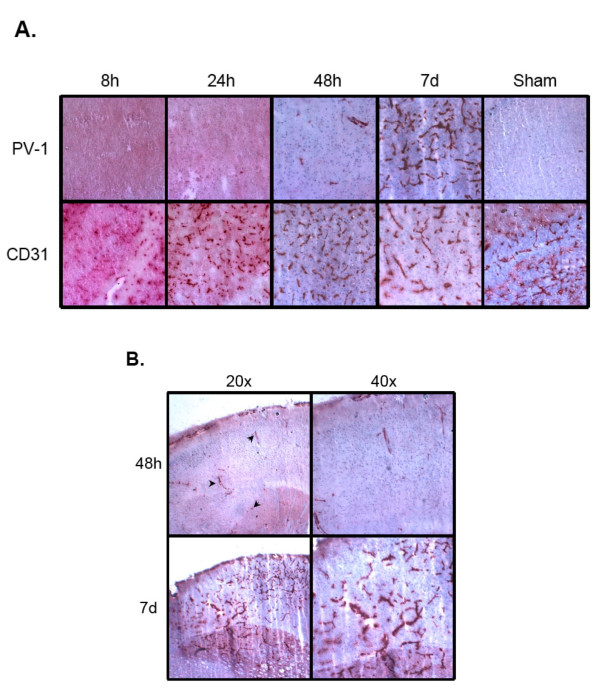
PV-1 protein expression after ischemic insult. A. Top row, PV-1; bottom row, CD31; 100×. PV-1 is not detected 8 or 24 hours after ischemia, unlike the endothelial control marker, CD31. PV-1 expression is detectable in a small number of vessels in the affected tissue 48 hours after injury. PV-1 protein is expressed in a majority of the vessels and is comparable to CD31 staining levels in the injured region by 7 days after injury. Sham (uninjured brain) shows no expression of PV-1. B. Overview of PV-1 expression within the ischemic region 48 h and 7d after injury. PV-1 is induced throughout the affected brain tissue at 48 h (see arrows) and remains evenly distributed by 7d.

**Figure 4:**
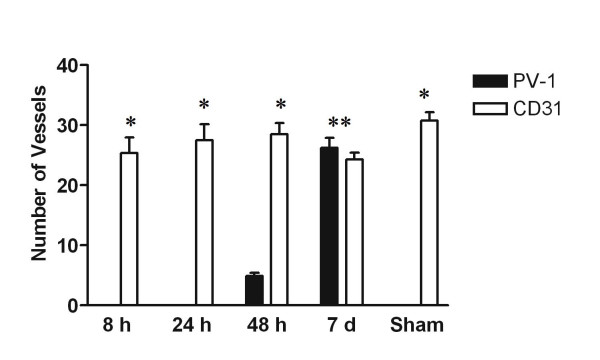
Vessel quantitation after ischemic insult. Dark bars, PV-1; open bars, CD31. Five high power fields were counted per mouse brain. Data are expressed as mean +/- standard error of the mean. *Statistically significant difference between the number of PV-1 positive and CD31 positive vessel (p < 0.001, 95% CI). **No statistically significant difference between number of PV-1 vessels and CD31 vessels (p > 0.05).

Previous studies in our laboratory have shown that *PV-1 *expression can also be upregulated in human patients who suffered from acute ischemia/stroke [6]. In order to demonstrate conservation of this response across species and to better characterize the timing of the response, we measured expression of PV-1 in acute ischemic mouse brains at 8 hours, 24 hours, 48 hours, and 7 days post-ischemia. Immunohistochemistry was performed on frozen brain sections to detect PV-1 and the positive control endothelial marker, CD31. As in normal brain, there was no PV-1 expression in the 8 hour or 24 hour post-ischemia mouse brain samples (Figure 3A). However, sparse PV-1 staining was present at 48 hours post ischemia, and dramatic upregulation was detected at 7 days post-ischemia, with PV-1 present in the majority of the vessels within the lesion. Induction of PV-1 occurred throughout the ischemic region and was not preferentially associated with any specific structure or domain (Figure 3B). All PV-1 staining was strictly localized to the area of ischemic damage, and not observed in the surrounding normal brain tissue. Mice that underwent sham surgeries did not express intracerebral PV-1. CD31 staining was positive for all samples (Figure 3A). Vessel quantitation confirmed that while no PV-1 was detected at 8 hours or 24 hours after injury, the number of PV-1 positive vessels increased to approximately 17% of CD31 expressing vessels by 48 hours. By 7 days post-ischemia, upregulation of PV-1 resulted in no statistical difference between the number of PV-1 positive vessels and CD31 positive vessels (Figure 4).

## Conclusion

The blood-brain barrier (BBB) plays a central role in maintaining fluid homeostasis in the brain. Microvascular endothelial cells are the anatomic basis of the BBB, which is comprised of both intercellular tight junctions that prevent paracellular transport as well as the tightly regulated facilitated transport systems that modulate intracellular transport. Loss of integrity of the BBB is a hallmark of numerous central nervous system pathologies, including malignant brain tumors and ischemic stroke. Failure of the BBB leads to increased fluid permeability in the cerebral microvasculature and extracellular fluid accumulation associated with vasogenic edema. This edema, due to leakage from abnormal tumor or ischemic blood vessels, generates increased intracranial pressure and contributes to neurologic deficits, secondary brain injury, herniation and, in the worst cases, death. However, while recent studies have advanced our understanding of the molecular mechanisms of the intact BBB, less is known about the endothelial gene expression changes associated with BBB breakdown.

Our previous studies of microvascular gene expression in glioblastoma identified *PV-1 *as a cell surface protein specifically upregulated in the endothelium of malignant brain tumors. In addition, we detected endothelial *PV-1 *re-expression in cases of acute ischemia. *PV-1 *was initially described as a microvesicular transport protein in the immature brain of rodents [4]. Induction of *PV-1 *expression stimulated the formation of stomatal diaphragms of caveolae, as well as transendothelial channels for microvesicular transport [8]. Inhibition of *PV-1 *expression using siRNA inhibited the expression of these structures associated with microvesicular transport [8]. While *PV-1 *is expressed normally in the adult lung, kidney and endocrine tissues of a rodent, its expression in the central nervous system is limited to the undifferentiated BBB and it is silenced in sites of intact BBB [4]. Re-expression of PV-1 suggests that this protein may play a functional role in BBB disruption and remodeling seen in tumors and after ischemic insults.

The development of effective vascular targeting strategies requires an improved understanding of the molecular changes that drive microvascular responses, as well as appropriate animal models in which to develop and test therapeutic regimens. However, our understanding of the relationship between human and rodent endothelial biology is still evolving. For example, impressive results using anti-angiogenic approaches in rodent trials are often not reproducible in humans [9]. This could be due, in part, to species-specific differences in the genes regulating endothelial phenotypes. Since PV-1 is a transmembrane protein, accessible via the bloodstream, it is an especially intriguing molecule. Characterization of *PV-1 *expression in a rodent model was therefore our next step towards the development of targeted therapy for glioma and stroke patients.

Intracranial U87:U87/VEGF mouse xenografts were created as a model for brain malignancy. The addition of the VEGF-overexpressing U87 cells (U87/VEGF) at the time of injection increased the vascular proliferation within the xenografts [7]. This made the xenografts more similar to human GBMs, which are highly vascularized, and facilitated analysis of gene expression within the tumor endothelium. However, we noted similar results using standard, unmodified U87MG intracranial xenografts as well. RT-PCR and quantitative RT-PCR of the resected mouse brains showed increased expression of *PV-1 *in the mouse xenografts in comparison to non-neoplastic mouse brains. Results were normalized to *flk-1 *to account for the increased vascularity of tumor versus normal brain tissue, thus ensuring that the increase in *PV-1 *expression was not simply due to an increase in the number of endothelial cells, but to tumor-related gene induction. This upregulation of *PV-1 *in the mouse xenograft parallels the increase in *PV-1 *expression seen in high grade human brain tumor samples [1,6]. The analyzed tumors were harvested between 28 and 34 days after intracranial injection of tumor cells. Attempts to investigate *PV-1 *expression at earlier times resulted in tumors that were undetectable or were small and poorly vascularized, leading to inconclusive results. Although larger, more stringent time course studies are required, this suggests that *PV-1 *is induced in large, well-vascularized tumors in mice, which is similar to the human state, where *PV-1 *is preferentially expressed in high-grade, well-vascularized astrocytomas (GBMs) than grade I/II astrocytomas [6].

Immunohistochemistry was performed to ensure protein expression was localized to the endothelium in the mouse xenografts (Figure 2). In addition, the endothelial expression of *PV-1 *was not generalized to the entire brain, but localized to the tumor. Non-neoplastic brain did not show any positive *PV-1 *staining, but did demonstrate CD31 reactivity. Additionally, *PV-1 *expression was detected using meca-32, which is a mouse-specific endothelial antibody. This indicates that the vasculature recruited to supply the tumor was of mouse origin and not human material.

Since preliminary studies in our laboratory have shown the upregulation of *PV-1 *in human acute ischemia samples, we next used an animal model of acute ischemia to determine if *PV-1 *was upregulated in ischemic mouse brain samples. In addition, the "on-off" nature of the MCAO injury makes it more amenable to time course studies than tumor models. While there was no upregulation of PV-1 at 8 hours or 24 hours post-ischemia, minimal PV-1 expression was detected 48 hours after the ischemic insult, followed by a dramatic increase in PV-1 expression 7 days after the ischemic injury. In all cases, detection of PV-1 was confined to the area of ischemia, and did not extend into the normal, uninjured brain. The delay in PV-1 expression may be attributed to the time required to synthesize the protein *de novo*. The observation that PV-1 is strongly expressed 7 days after injury also suggests that this protein may be associated with revascularization of the damaged tissue and the protein may be induced in new, immature microvessels, similar to those found in aggressive tumors [10,11].

So while *PV-1 *expression in the cerebrovasculature is completely suppressed by the intact, differentiated BBB, it is dramatically induced during states of BBB disruption such as malignancy and ischemia in both human and rodent brain. The progression and recurrence of malignant brain tumors is associated with the expression of pro-angiogenic growth factors, such as VEGF and SF/HGF (reviewed in [12,13]). Tumor associated angiogenesis results in markedly abnormal vessels [14,15]. These vessels are poorly organized, irregular and tortuous. Furthermore, the new vessels tend to be leaky and hemorrhagic, partly due to induction of VEGF, with disrupted cell-cell junctions and loosely adherent or absent pericytes [16]. This leads to failure of the BBB integrity and contributes to vasogenic edema in advanced brain tumors. Similarly, hypoxia has been shown to induce expression of VEGF 48 hours to 72 hours after ischemic insult and has been linked to induction of neoangiogenesis by 3 days post injury [17,18]. Hypoxia-induced VEGF expression after cerebral ischemic insult precedes edema while VEGF antagonism can reduce the post-ischemic edema and associated tissue damage [18,19]. We, and others, have shown that exogenous VEGF induces the expression of PV-1 in cultured endothelial cells [3,6]. PV-1 expression has also been correlated with microvascular leakage in diabetic retinopathy, indicative of a role for this protein in disruption of the blood-occular barrier [20]. Re-expression of PV-1, a cell surface protein with a putative function in transendothelial transport, in brain malignancy and acute ischemia, suggests that this protein may play an active role in BBB leakiness. The characterization of *PV-1 *expression in murine models could be a first step towards development of *PV-1 *targeted strategies.

## Methods

### Cell Culture

U87MG tumor cells (ATCC, Manassas, VA) were maintained in Minimum Essential Medium Eagle with Earle's Salts and L-glutamine (Invitrogen, Carlsbad, CA), supplemented with 10% FBS, 0.1 mM MEM non-essential amino acids, 0.1 mM MEM sodium pyruvate, sodium bicarbonate, and antibiotics. U87MG/VEGF_165 _cells (gift of Dr. Shiyuan Cheng, University of Pittsburgh) were maintained in Dulbecco's Modified Eagle's Medium (Invitrogen) supplemented with 10% FBS and antibiotics.

### RNA Isolation and Reverse Transcription

Tumors were dissected free from non-neoplastic tissue. RNA was isolated from 10–30 mg of frozen, homogenized tumor or normal brain from a control mouse using the Qiagen RNeasy Mini Kit (Qiagen, Valencia, CA). cDNA was generated using the SuperScript First-Strand Synthesis System (Invitrogen). Briefly, 5 μg of total RNA was combined with oligo(dT)_12–18 _primers, heat denatured and reverse transcribed with SuperScript II RT. An RT-minus control was performed for each reverse transcription reaction. Equivalent volumes of RT-plus and RT-minus products were used as templates for amplification.

### PCR

Primers were synthesized by IDT (Coralville, IA). Primer sequences were PV1(F) 5'-gccagaagttggagctagagcg-3', PV1(R) 5'-ggagcagagtgataggaaaccg-3', FLK1(F) 5'-gccagggcaaggactacg-3', FLK1(R) 5'-ggtagtgtagtcaggagcccg-3'. Standard reaction conditions were 1× PCR buffer, 200 mM dNTPs (Invitrogen), 4% DMSO (Sigma), 1.5 mM MgCl_2_, 1 μM sense primer, 1 μM antisense primer, 1.5 units Platinum Taq (Invitrogen), and 2 μl cDNA in a total reaction volume of 25 μl. Reactions were overlaid with mineral oil and cycled in a PTC-200 thermalcycler (MJ Research, Waltham, MA).

### Quantitative RT-PCR

qRT-PCR was carried out on triplicate samples for 40 cycles of 10s at 95°C, 30s at 60°C after an initial incubation at 95°C for 3 min in an Chromo4 thermal cycler (Bio-Rad, Hercules, CA). Reaction conditions were 1× iQ SYBR Green super mix (Bio-Rad), 400 nM forward and reverse primers, and 2.0 μl cDNA in a total reaction volume of 25 μl. Amplification of *flk-1 *was performed for each cDNA in triplicate for normalization of RNA content. Primer sequences were: PV1q(F) 5'-gggtggttggaaatgatactgg-3', PV1q(R) 5'-cacgatgccatgctggtcac-3', FLK1q(F) 5'-ttccgccagtgccaagg-3', FLK1q(R) 5'-gccacctccatctccagtgtc-3'. Threshold cycle number (*Ct*) of amplification in each sample was determined by Bio-Rad software. Relative mRNA abundance was calculated as the *Ct *for amplification of *PV-1 *minus average *Ct *for *flk-1*, expressed as a power of 2, *i.e*. 2^-ΔCt^. Three individual *PV-1 *values were averaged to obtain mean ± SD. Statistical significance was determined using a Wilcoxon rank sum test.

### Intracranial xenografts

U87MG cells were harvested with trypsin (Invitrogen) and resuspended at a concentration of 10^5 ^cells/10 μl antibiotic-free DMEM. For increased vascularity, U87MG/VEGF_165 _cells and U87MG cells were harvested and resuspended at a 1:5 ratio for a final concentration of 10^5 ^cells/10 μl antibiotic-free DMEM (U87:U87/VEGF tumors). Athymic nu/nu mice (NCI, Frederick, MD) were anesthetized using an intraperitoneal injection of 3 ml/kg xylazine/ketamine. The cranium of each anesthetized mouse was fixed in a stereotactic frame fitted with a small animal adaptor. A 1.5 mm burr hole was made in the cranium exactly 2.5 mm lateral to bregma. A 26 g beveled tip Hamilton syringe was used to inject 10 μl of tumor cell solution into the posterior striatum of the mouse. Animals demonstrating symptoms of disease were euthanized by barbiturate overdose followed by decapitation, 28–34 days post-inoculation. Brains were surgically removed and snap frozen. For RNA isolation, tumors were dissected free from non-neoplastic brain. For immunohistochemistry, whole brains were sectioned.

### Cerebral ischemia

Focal cerebral ischemia was produced by intraluminal occlusion of the left middle cerebral artery (MCAO) with a nylon monofilament suture as previously described [21-23]. In brief, male 2- to 3-month-old C57/B6 mice (The Jackson Laboratory, Bar Harbor, ME) were anesthetized with 1.5% isoflurane in a 30%0_2_/68.5% N_2_O mixture under spontaneous breathing. Systolic arterial blood pressure was monitored through a tail cuff (XBP1000 Systems, Kent Scientific Corporation, Torrington, CA) and arterial blood gas was analyzed at 15 min before and 30 min after the onset of ischemia. The animals underwent MCAO for 90 minutes. Changes in regional blood flow were evaluated before, during and after MCAO using laser Doppler flowmetry. Brains were harvested at the indicated times after surgery and snap frozen.

### Antibodies

Anti-PV-1 (meca-32) rat polyclonal antibody and anti-CD31 rat polyclonal antibody were from BD Biosciences Pharmingen (San Jose, CA). Biotinylated rabbit-anti-rat secondary antibody was from Vector Laboratories (Burlingame, CA).

### Immunohistochemistry

Brains were frozen in OCT and sliced through the tumor into 10 μm coronal sections. Sections were fixed in -20°C acetone for 15 minutes. Slides were washed in 1× PBS and then quenched with 0.3% H_2_0_2 _in 0.3% normal serum/1× PBS for 5 minutes. Slides were washed in 1× PBS then blocked for 30 minutes with 4% normal rabbit serum/1xPBS. Sections were exposed to anti-PV-1 (1:100) or anti-CD31 (1:500) at 4°C overnight. Sections were washed with 1× PBS and exposed for 1 hr to biotinylated rabbit-anti-rat secondary, diluted 1:1000 for PV-1 and 1:2500 for CD31. Sections were washed in 1× PBS then exposed to Vectastain ABC (Vector) for 30 minutes. Sections were washed in 1× PBS and incubated with NovaRed substrate (Vector) for 15 minutes. Slides were rinsed with distilled water and counterstained with Mayer's Hematoxylin (Biomeda, Foster City, CA). Sections were rinsed with distilled water, exposed to 0.08% NH_4_OH for 3 minutes, then rinsed again with distilled water. Crystal Mount (Biomeda) was applied to slides and allowed to dry overnight.

### Vessel Quantitation

Ischemic and control brain sections were immunostained for PV-1 or CD31, as described above. Two lesioned or control animals were counted for each protein at 8 h, 24 h, 48 h and 7d post-infarct (four animals per time point), with the exception of 8 h, where one unique animal was analyzed for each stain. The number of vessels in five non-overlapping, representative fields within the ischemic (both core and penumbra) or corresponding control zone was counted at 400× magnification on each slide. Statistical significance was determined by two-way ANOVA analysis.

## Abbreviations

VEGF, vascular endothelial growth factor; PDGF, platelet derived growth factor; SF/HGF, scatter factor/hepatocyte growth factor; RT-PCR, reverse transcriptase polymerase chain reaction

## Authors' contributions

EHS performed immunohistochemical studies and assisted with manuscript preparation. EBC-W performed PCR experiments, and assisted with study design and preparation of the manuscript. BNW participated in xenograft experiments and immunohistochemistry. YL performed quantitative RT-PCR analysis. ZSA participated in ischemia experiments and statistical analysis. JC performed the ischemia surgeries and provided experimental design advice. KAW directed the study and manuscript preparation. All authors read and approved the final manuscript.
